# Random and independent sampling of endogenous tryptic peptides from normal human EDTA plasma by liquid chromatography micro electrospray ionization and tandem mass spectrometry

**DOI:** 10.1186/s12014-017-9176-7

**Published:** 2017-12-08

**Authors:** Jaimie Dufresne, Angelique Florentinus-Mefailoski, Juliet Ajambo, Ammara Ferwa, Peter Bowden, John Marshall

**Affiliations:** 1 Ryerson University, 350 Victoria Street, Toronto, ON M5B 2K3 Canada; 2 Integrated BioBank of Luxembourg, 6 r. Nicolas-Ernest Barblé, Dudelange, 1210 Luxembourg

## Abstract

**Background:**

Normal human EDTA plasma samples were collected on ice, processed ice cold, and stored in a freezer at – 80 °C prior to experiments. Plasma test samples from the – 80 °C freezer were thawed on ice or intentionally warmed to room temperature.

**Methods:**

Protein content was measured by CBBR binding and the release of alcohol soluble amines by the Cd ninhydrin assay. Plasma peptides released over time were collected over C18 for random and independent sampling by liquid chromatography micro electrospray ionization and tandem mass spectrometry (LC–ESI–MS/MS) and correlated with X!TANDEM.

**Results:**

Fully tryptic peptides by X!TANDEM returned a similar set of proteins, but was more computationally efficient, than “no enzyme” correlations. Plasma samples maintained on ice, or ice with a cocktail of protease inhibitors, showed lower background amounts of plasma peptides compared to samples incubated at room temperature. Regression analysis indicated that warming plasma to room temperature, versus ice cold, resulted in a ~ twofold increase in the frequency of peptide identification over hours–days of incubation at room temperature. The type I error rate of the protein identification from the X!TANDEM algorithm combined was estimated to be low compared to a null model of computer generated random MS/MS spectra.

**Conclusion:**

The peptides of human plasma were identified and quantified with low error rates by random and independent sampling that revealed 1000s of peptides from hundreds of human plasma proteins from endogenous tryptic peptides.

**Electronic supplementary material:**

The online version of this article (10.1186/s12014-017-9176-7) contains supplementary material, which is available to authorized users.

## Background

The analysis of proteins and peptides from human blood by liquid chromatography, electrospray ionization and tandem mass spectrometry (LC–ESI–MS/MS) may permit the diagnosis of diseases and the evaluation of the efficacy of therapeutic treatments [1]. Both MALDI and electrospray ionization methods previously demonstrated that tryptic protease activities cleave peptides from blood proteins that differed between control and heart attack samples [2, 3]. At least some peptides observed in serum samples are known to be ex vivo artefacts that represent the steady-state balance of endo- versus exopeptidase action after sample collection [2, 4]. The peptides of blood are in a dynamic steady state that can be easily perturbed by sample incubation conditions [2, 4]. Artefacts introduced into the blood fluids after sampling and prior to freezing may be a major source of pre-analytical variation [2, 5–21]. There is considerable variation in the peptides observed, and even the trends reported, in the study of degradation of blood proteins that may differ between groups, likely from the large variation that occurs immediately after sample collection [2, 5–21]. One key contribution of this study is the collection of EDTA plasma directly onto ice for cold processing to establish a reliable baseline compared to plasma peptides at room temperature.

There are many potential sources of error in the identification and quantification of peptides and therefore proteins [22]. Collecting rich MS/MS spectra at a high signal-to-noise is the key to both reliable and sensitive identification and quantification of blood peptides and proteins with the X!TANDEM algorithm [23, 24]. It is necessary to pre-fractionate samples to prevent co-elution of peptides with similar m/z values. Selective extraction from the solid phase with organic aqueous solvent is a simple means to enrich endogenous peptides from blood fluids [2–4, 25]. Robust and sensitive ion traps may have advantages in analyzing low amounts of blood peptides and proteins where signal-to-noise filters and statistical controls are employed to control type I error rate [26–28]. The X!TANDEM algorithm is known to fit the MS/MS spectra from sensitive and robust ion traps with a high degree of statistical rigour using the default settings of ± 3 m/z of the precursor and ± 0.5 Da of the many fragments with up to three missed cleavages [29] with a low rate of type I error in the identification of peptides based on the goodness of fit of the MS/MS spectra [23, 24, 30, 31]. The proteins of human plasma contain many highly abundant proteins such as albumin, apolipoproteins, protease inhibitors and others that digest efficiently with the exogenous addition of trypsin and thus mask the detection of low abundance proteins by LC–ESI–MS/MS [1]. Many of the well-known proteins of human blood [32, 33] are cleaved by endogenous tryptic endopeptidases to release fully tryptic peptides that may be identified by collection over C18 followed by LC–ESI–MS/MS with an ion trap [3]. The peptides of blood fluids show good agreement on the detection of many unexpected cellular peptides by LC–ESI–MS/MS using both Qq-TOF and the sensitive ion trap [2–4, 25, 34]. The secretion or release of cellular proteins into extracellular space may result in the preferential cleavage of the cellular proteins upon exposure to circulating protease activities [35]. Here, the random and independent sampling of endogenous tryptic peptides were compared from plasma collected and incubated on ice versus the same plasma incubated at room temperature and showed many plasma proteins are degraded over time by tryptic proteases with about a twofold higher frequency of many tryptic peptides at room temperature compared to ice cold samples. A second key contribution of this paper is to show that the peptides from proteins expressed in tissues and cells may be identified and quantified by micro electrospray with LC–ESI–MS/MS of endogenous peptides with a simple linear ion trap [36] that shows a low type I error rate by comparison to a null model of random MS/MS spectra, or noise spectra, and computing protein p-values using X!TANDEM that shows a low False Discovery Rate (low q-values) using the method of Benjamini and Hochberg [37].

## Methods

### Materials

The Agilent 1100 HPLC (Santa Clara, CA, USA) was coupled to an XL LTQ linear ion trap mass spectrometer (Thermo Electron Corporation, Madison, WI, USA) for LC–ESI–MS/MS. The protease inhibitors, salts, buffers, Coomassie Blue and ninhydrin were obtained from Sigma Aldrich (St. Louis, MO, USA). The #1 filter paper was obtained from Whatman (Maidstone, UK). The HPLC grade water and acetonitrile were obtained from Caledon Laboratories (Georgetown, ON, Canada).

### Plasma sample collection

Human plasma was collected under a Comité National d’Ethique de Recherche (CNER) Protocol #201107 “Biospecimen Research” at the Centre Hospitalier de Luxembourg. The plasma was collected in EDTA tubes (Becton Dickenson) that were rapidly rotated 5 times before packing in ice. The ice cold plasma was then separated from blood cells at 12,000 RCF for 20 min at 4 °C prior to aliquoting to 225 µl samples on ice and randomly assigned to short term or long term experimental treatments. Plasma samples of 225 μl were previously shown to be sufficient for peptide extraction [4, 25, 34].

### Plasma sample treatments

Random aliquots were maintained on ice, ice plus protease inhibitors, or incubated at room temperature for ≥ 96 h or more as indicated. Plasma samples were permitted to degrade at room temperature for 0 h (ICE), 1, 4, 8, 12, 24, 48 and 72 h alongside ice or ice plus inhibitor controls prior to random sampling and analysis. A total of 82 control plasma samples that were either frozen or never above ice cold (ALLICE), and 88 plasma samples that were incubated at room temperature for different lengths of time (ALLRT), were analyzed. The protease inhibitor treatment consisted of Sigma Eukaryotic protease inhibitor cocktail plus 2 mM AEBSF, 2 mM PMSF, 2 mM EDTA, 2 mM caproic acid, and 2 mM benzamidine. The Sigma Mammalian Protease inhibitor cocktail (contains at least: AEBSF, 104 mM, Aprotinin, 80 μM, Bestatin, 4 mM, E-64, 1.4 mM, Leupeptin, 2 mM, Pepstatin A, 1.5 mM) was used at 1/100 (v/v). Plasma samples (225 µl) from at least 10 different donors were tested at each time point and over the time course of degradation up to 72 h. At the end of each time period, the samples were frozen, freeze dried and stored dried at − 80 °C until analysis.

### Protein assay, SDS-PAGE and free amine assays

Protein content of the plasma samples was determined in the presence of SDS detergent by the Dumbroff method [38] prior to separation by tricine SDS-PAGE followed by staining of the gel with Coomassie Brilliant Blue [39]. Plasma samples were mixed 1:1 with 2 × tris sample buffer for SDS-PAGE and then diluted a further 10 fold in 2 ×  tris sample buffer into the linear BSA standard range for dot blot protein assays. The free amines were extracted in ethanol and measured using the Cd-ninhiydrin peptidase assay alongside glycine equivalents [40].

### Unbiased LC–ESI–MS/MS

The mass spectrometer was cleaned, calibrated with the manufacturer’s standard mixture, tuned with GluFib and Angiotensin and tested for sensitivity prior to each replicate block by infusion of a dilution series of GluFib and Angiotensin. The mass accuracy and sensitivity of the LC–ESI–MS/MS system was tested with a tryptic digestion of a mixture of cytochrome C, glycogen phosphorylase B and alcohol dehydrogenase [26]. The plasma peptides were collected over a C18 preparative column with elution in 2 µl of 5% formic acid and 65% acetonitrile and immediately diluted with 18 µl of 5% formic acid for injections via a 20 µl loop with a Rheodyne manual injector. A total of ~ 5 µg of extracted and purified peptides was injected for each analytical HPLC separation over a 300 micron ID column (15 cm) with inline filter frits. The peptides were ionized at 4.5 kV via a micro electrospray ion source with 10 L N_2_ per minute with a transfer capillary temperature of 200 °C into a Thermo Electron Corporation LTQ ion trap mass spectrometer [36]. The peptides were randomly and independently sampled by MS and MS/MS without replacement as the peptides eluted from the HPLC column into the electrospray source from 350 to 2000 m/z.

### Peptide MS/MS spectra correlation analysis

A federated library of 158,072 human proteins that differed by at least one amino acid was assembled from NCBI, Ensembl and Swiss Prot and made non-redundant using Structured Query Language (SQL). A physical filter of at least one thousand (E3) intensity counts for peptide parent ions was used to limit type I error [23, 24]. A sub-set of the data was analyzed by fully tryptic versus no enzyme specified to compare the sets of proteins identified. The MS and MS/MS spectra of all peptides recorded were correlated to the federated library set with fully tryptic enzyme specification, a charge state of 2^+^ or 3^+^ with up to three missed cleavages in SEQUEST [41, 42], MASCOT [43], OMSSA [44] and X!TANDEM [29] with ± 3 m/z for the precursor and with the fragments within 0.5 Da with up to three missed cleavage sites [23, 24, 26, 27, 30, 45]. The data from SEQUEST, provided via the manufacturer’s BIOWORKS algorithm, was further limited to the default setting of 0.05 maximum delta correlation of parent ions with maximum peptide mass set to 5000 Da and peptide length set to ≥ 6 amino acids. The authentic results were compared to random mis-correlations from unexpected modifications using computer generated random MS/MS spectra or from noise spectra by blank runs with HPLC grade solvents over naïve columns.

### Computational analysis in SQL and statistical analysis with R

The combination of Chi Square and general linear models such as regression and ANOVA using a generic statistical analysis system are sufficient to provide a satisfying statistical analysis of LC–ESI–MS/MS data [26–28, 45]. The resulting peptide and protein identifications together with the parent and fragment m/z and intensity values were parsed into an SQL database [30]. The charge state with the best score (Filter 1) and the peptide sequence with the best score (Filter 2) were used to control type I error of identification: thus, the SQL database utilized a unique hash tag for each MS–MS/MS event to ensure that only the best fit of each MS/MS spectra at only one charge state was accepted, and thus no MS/MS spectra was assigned to more than one peptide sequence. The peptide-to-protein counts of plasma samples were previously statistically analyzed in the generic statistical system S [2] or SAS [23, 24, 26, 27, 30, 31, 45]. However, in this study the data was analyzed using the generic open-source R statistical system [46]. The R statistical system was also used to plot the peptide-to-protein distribution of authentic peptides compared to those of random spectra and to compute the cumulative p-value for protein Gene Symbols from the product of the observed peptides p-values from the best accession [30].

## Results

EDTA plasma collected directly onto ice were analyzed by C18 preparative chromatography followed by micro electrospray LC–ESI–MS/MS of samples incubated on ice or ice plus protease inhibitors. Samples that remained on ice showed about half of the endogenous tryptic peptides of samples incubated at room temperature, and thus many or most of the peptides observed in EDTA plasma over time are ex vivo artefacts.

### Tricine SDS-PAGE

**Fig. 1:**
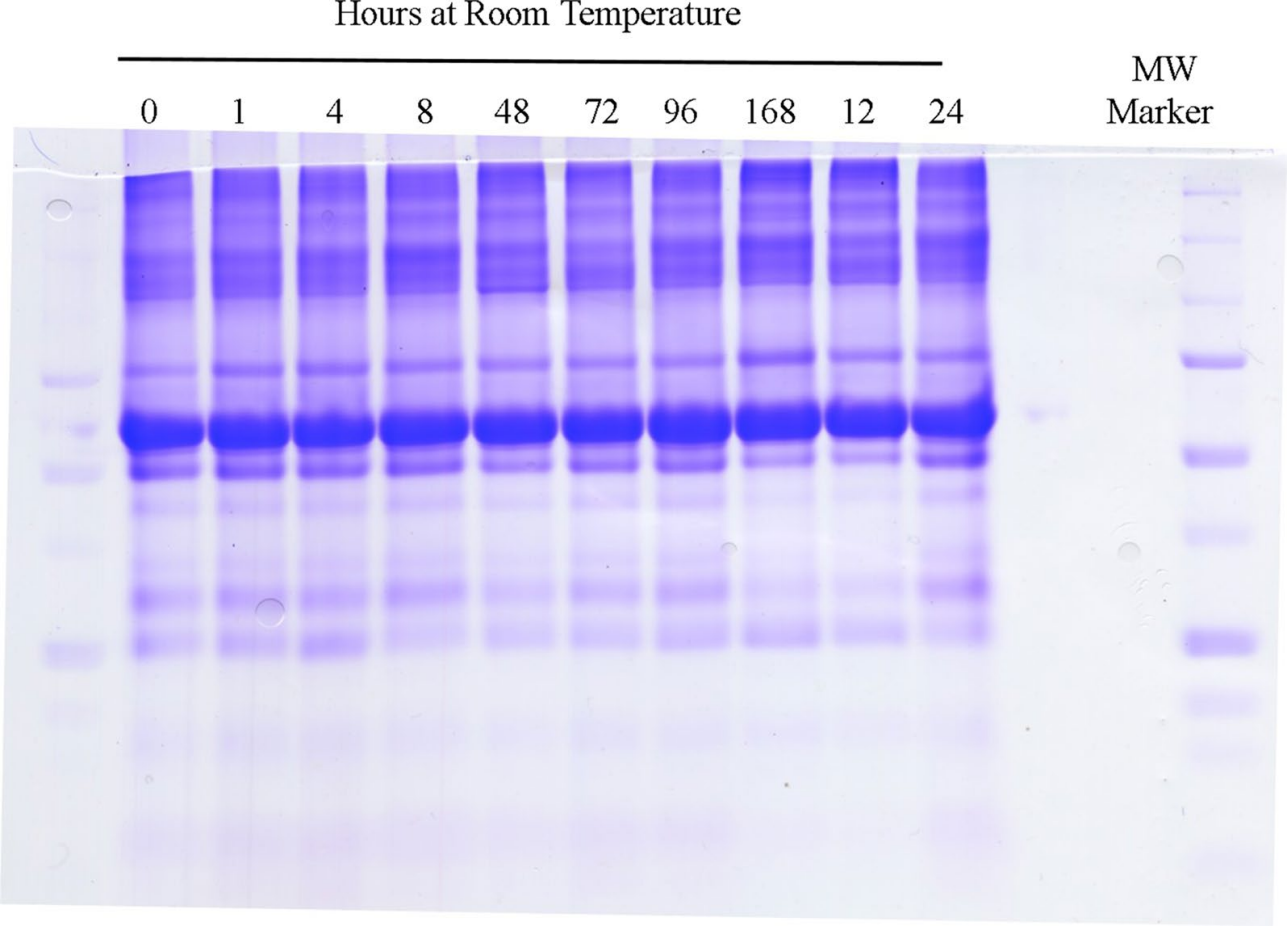
The stability of EDTA plasma as demonstrated by electrophoresis and staining with Coomassie Brilliant Blue. EDTA Plasma samples were diluted 1:1 with 2 × tris sample buffer for tricine SDS-PAGE and 4 µL loaded per lane (~ 100 µg). Molecular markers 250, 150, 100, 75, 50, 37, 25, 15 kDa. The tricine SDS-PAGE gel shown is representative of four technical replicates

Tricine SDS-PAGE showed no obvious protein degradation and no new bands were observed with time at room temperature (Fig. 1). The degradation of the plasma samples at room temperature for up to 96 h or more was not detectable by separation of ~ 100 µg of plasma proteins by tricine SDS-PAGE with staining by CBBR [2, 47]. The major protein bands of plasma from SDS-PAGE appear to be stable with incubation at room temperature even for many days and so do not appear to degrade much.

### Protein assay

**Fig. 2:**
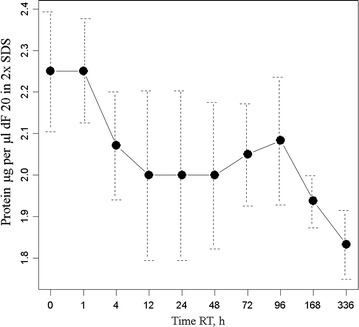
Total protein analysis by the Dumbroff method of non-covalent binding to filter paper followed by washing of non-proteinaceous components with methanol prior to staining with 0.1 g CBBR_250_ in 40% methanol and 10% acetic acid. Plasma samples were mixed 1:1 with 2 × tris sample buffer for SDS-PAGE and then diluted a further 10 fold in 2 × tris sample buffer (dF 20) into the linear BSA standard range

The protein content of the blood plasma samples in SDS-PAGE sample buffer were measured by the Dumbroff method [38] and was on average about 45 mg/ml but showed great variability within the randomly chosen samples. The total protein assays of the samples showed that there was a slight decline of about 10% in the protein content over the course of the 24 h at room temperature (Fig. 2). Protein content was too variable between individuals to be a useful measure of degradation but seems to indicate that some proteins were susceptible to degradation in the sample.

### Free amine assay

**Fig. 3:**
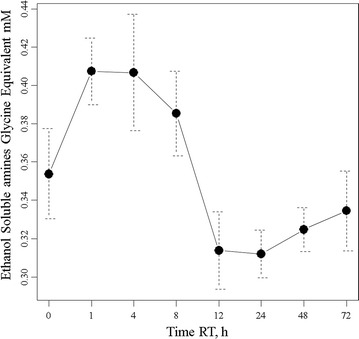
The release of ethanol-soluble free amines from human EDTA plasma over time at room temperature. Dried plasma (225 µL) was extracted with 250 µL of absolute ethanol prior to reaction with Cd-ninhydrin alongside glycine standards

The modified colorimetric (Cd-ninhydrin) method to detect peptidase activity by the release of free amines is sensitive to micromole amounts of released free amines [40]. The free amine assay showed that there is a rapid and statistically significant release of alcohol-soluble amines over time from 350 to about 410 micro molar soon after incubation at room temperature (Fig. 3). The free amine results were consistent with the protein assay that shows large variation between individuals but also an apparent release of ethanol soluble amines early in the incubation of plasma at room temperature.

### Random and independent sampling

A random and independent sampling of endogenous peptides from EDTA plasma maintained on ice versus at room temperature was made without replacement as the peptides eluted from the C18 HPLC column in the LC–ESI–MS/MS system. A total of 563,095 MS/MS spectra from precursors with intensity ≥ 1000 counts were obtained from LC–ESI–MS/MS analysis of 170 samples × 90 min C18 HPLC runs. The LC–ESI–MS/MS spectra and the results of the correlation algorithms were parsed into an SQL Server Database for statistical analysis with the generic statistical analysis system R [23, 24, 30, 31, 48].

### Fully tryptic versus no enzyme correlation

**Fig. 4:**
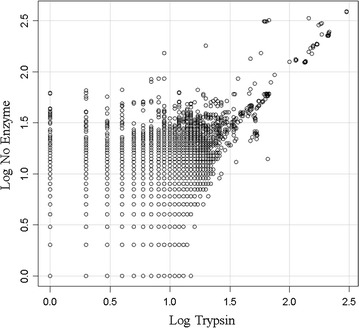
The comparison of the fully tryptic peptide correlation with up to three missed cleavage sites versus no enzyme specification. All proteins with at least 1 peptide correlation under either condition were plotted. The results indicate that the same set of proteins are correlated with no enzyme versus trypsin but that more peptides per protein are detected with the computationally more intense no enzyme parameter

Comparing the set of proteins identified by fully tryptic correlation versus no enzyme correlation (all possible human peptide sequences) showed essentially complete agreement on the set of proteins identified (Fig. 4). The no-enzyme search returned a greater number of peptides to the same small group of proteins identified by tryptic correlation and did not identify many new proteins from the much larger set of proteins not identified by tryptic correlation.

### Filter by best charge state and peptide sequence

The MS/MS spectra were then correlated to 158,072 federated protein sequences by the MASCOT, OMSSA, X!TANDEM and SEQUEST algorithms. The results of the LC–ESI–MS/MS from the fully tryptic correlation algorithms were parsed together into an SQL Server Database for statistical analysis with the R generic statistical analysis system [23, 24, 30, 31, 48]. A major source of error in the results of LC–ESI–MS/MS analysis was the re-use of MS/MS spectra for more than one protein by MASCOT, OMSSA, SEQUEST and X!TANDEM (Table 1). Filtering out all hits that were not the best charge state, i.e. 2^+^ or 3^+^ (Filter 1) and then accepting only the best peptide sequence at the best charge state (Filter 2) eliminates more than 80% of the potential peptide correlations (Table 1). A total of 563,095 MS/MS spectra from parent ions ≥ E3 (1000) counts resulted in correlations to 729,533 peptides from a library of 158,072 proteins. A sum total of 3,788,530 peptides were correlated by the algorithms together over all treatments. After selecting only the best fit in terms of charge state and peptide sequence where the parent protein showed at least three independent correlations, the number of peptides and proteins collapsed to 729,533 peptides from 82,276 protein accessions.

**Table 1:** The summary of the total number of peptide correlations and the number of protein accession numbers detected from a total of 563,095 MS/MS spectra from human EDTA plasma from the sum of MASCOT, OMSSA, X!TANDEM and SEQUEST against a library of 158,072 human protein accessions with respect to filter conditions in an SQL Server Database

Filter	Total correlations	Number of protein accessions
All	5,414,868	401,960
Filter 0	3,788,530	150,678
Filter 1	833,029	125,641
Filter 2	793,309	125,641
Filter 2	729,533	81,276
≥ 3 peptides		
Per accession		

The results of 170 samples collected and maintained ice cold or preserved (82) versus samples incubated at room temperature for up to 72 h (88) prior to C18 solid phase extraction and micro LC–ESI–MS/MS with a linear ion trap are shown

### Correlation algorithms

**Fig. 5:**
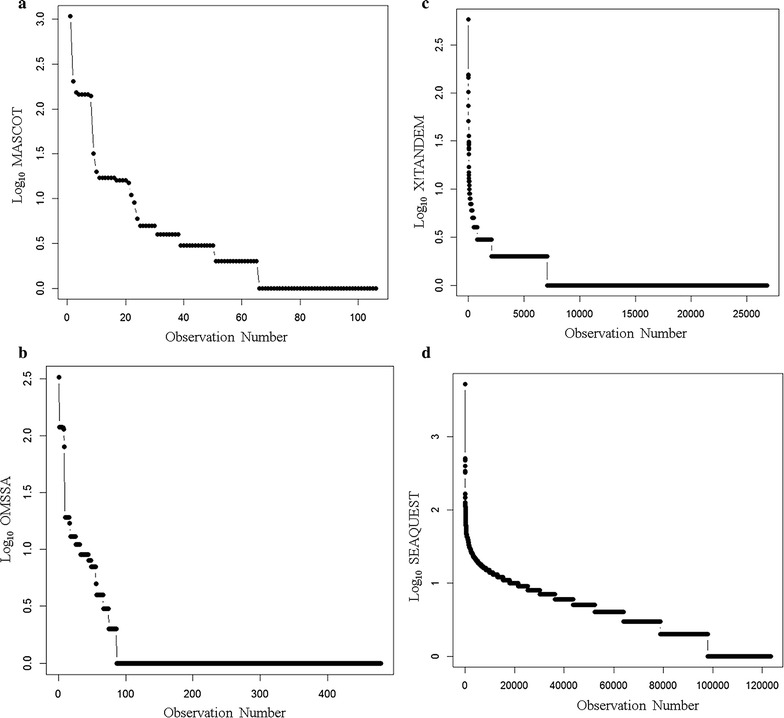
The comparison of the results of MASCOT, OMSSA, X!TANDEM and SEQUEST algorithms for the identification of endogenous tryptic peptides from human plasma on ice and incubated at room temperature combined. The y axis shows the log_10_ peptide correlation per protein accession from the MASCOT, OMSSA, X!TANDEM and SEQUEST. Panels **a** the log_10_ peptide correlation per protein accession from the MASCOT algorithm; **b** the log_10_ peptide correlation per protein accession from the OMSSA algorithm; **c** the log_10_ peptide correlation per protein accession from the X!TANDEM algorithm; **d** the log_10_ peptide correlation per protein accession from the SEQUEST algorithm

**Fig. 6:**
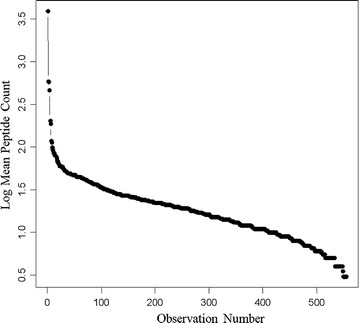
The total peptide-to-protein counts for all algorithms and all treatments. A total of 563,095 MS/MS spectra were obtained from LC–ESI–MS/MS analysis of 170 samples × 90 min HPLC runs over C18 300 micron × 150 mm were computed

From a total of 563,095 MS/MS spectra of greater than 1000 arbitrary counts, MASCOT fitted just over 60 proteins, OMSSA fitted 74 proteins, X!TANDEM fitted over 2068 protein sequences, and SEQUEST fitted 78,929 proteins that differ by at least one amino acid from the federated library of all known protein forms with at least three peptides (Fig. 5). Most of the peptides and proteins identified by X!TANDEM were further identified by SEQUEST. The peptides of normal human plasma extracted by C18 solid phase extraction and analyzed by micro electrospray are listed by accession number in the Additional file 1. Plotting the best fit data in terms of the averaged peptides to proteins from MASCOT, OMSSA, X!TANDEM and SEQUEST shows that some proteins show more than a hundred independent best fit correlations (Fig. 6). Since X!TANDEM previously showed a low type I error rate for proteins from fully tryptic peptides [23, 24, 26, 27] and since the goodness of fit of MS/MS spectra also sensitively identified far more proteins than either MASCOT or OMSSA, X!TANDEM was selected for subsequent statistical analysis. X!TANDEM fit some 583,927 random and independently sampled MS/MS spectra that showed 48,019 correlations to a set of 5855 peptides from 2068 proteins that reduced to 510 Gene Symbols with at least 5 peptides that had precursor intensity values greater than 1000 counts (E3). The MASCOT and X!TANDEM algorithms have been compared [49], in agreement with previous results we observed that under our conditions SEQUEST was the most sensitive, X!TANDEM was sensitive and MASCOT and OMSSA were less sensitive [50]. Thus under these conditions MASCOT and OMSSA showed few proteins compared to X!TANDEM, that is known to be reliable compared to random simulations, and thus showed a large type II error.

### Incubation treatments

Collecting EDTA plasma samples directly onto ice, followed by incubation on ice ± inhibitors, resulted in about 20,000 total peptide correlations per LC–ESI–MS/MS run but also prevented the generation of endogenous plasma peptides for days on ice especially if protease inhibitors were provided and so yields a stable baseline for degradation experiments. In contrast, samples that were collected on ice and then briefly incubated at room temperature showed an increase to about 40,000 thousand peptide MS/MS correlations per run by 1 h at room temperature (Table 2). The samples that were freeze dried and stored at room temperature or freeze dried and stored at − 20 °C both had about 20,000 thousand peptide correlations per LC–ESI–MS/MS run similar to samples collected and stored briefly on ice. Samples preserved by freezing at − 80 °C or liquid nitrogen showed slightly higher levels of peptides per run than ice or freeze drying (Table 2).

**Table 2:** The sum peptide-to-protein corrections per LC–ESI–MS/MS run from the MASCOT, OMSSA, X!TANDEM and SEQUEST algorithms over the experimental treatments of preserved and degraded human EDTA plasma

TreatmentName	Sum.Correlations	N	CorrelationsPerRun
FD-20 °C	346,290	12	28,858
− 80 °C	254,832	7	36,405
LN	401,209	11	36,474
FDRT	280,050	12	23,338
ICE_INHIB_1 h	42,197	2	21,099
ICE_INHIB_4 h	44,270	2	22,135
ICE_INHIB_8 h	90,814	4	22,704
ICE_INHIB_24 h	102,271	4	25,568
ICE_INHIB_48 h	80,709	4	20,177
ICE_INHIB_72 h	77,590	3	25,863
ICE_1 h	49,197	2	24,599
ICE_4 h	74,232	3	24,744
ICE_8 h	66,377	2	33,189
ICE_24 h	115,975	4	28,994
ICE_48 h	107,433	5	21,487
ICE_72 h	113,023	3	37,674
RT_1 h	538,873	12	44,906
RT_4 h	351,457	12	29,288
RT_8 h	327,895	10	32,790
RT_12 h	435,866	12	36,322
RT_24 h	507,283	12	42,274
RT_48 h	349,692	10	34,969
RT_72 h	403,881	11	36,716
RT_96 h	227,777	6	37,963

The redundant correlations made by the sum of all four algorithms to any human protein are shown. Abbreviations: FD-20 °C, Freeze Dried and stored in an electric freezer at − 20 °C for 1 year; − 80 °C, fresh plasma stored in an electric freezer at – 80 °C for 1 year; LN, fresh plasma stored in liquid nitrogen for 1 year; FDRT, freeze dried and stored at room temperature for 1 year; ICE_INHIB, fresh plasma stored on ice with a cocktail of protease inhibitors; ICE, fresh plasma stored on ice; RT, room temperature

### Analysis of ice versus room temperature samples

**Fig. 7:**
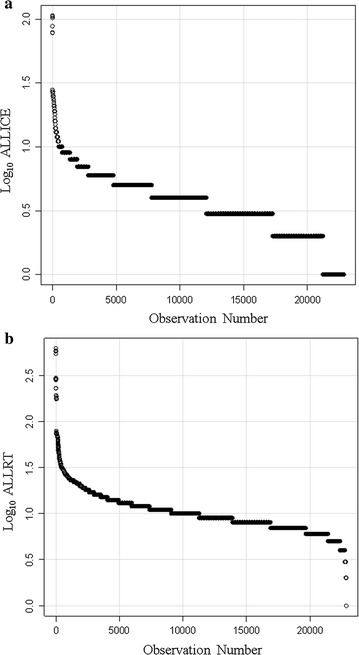
The log peptide-to-protein count of the preserved and degraded peptides of human EDTA plasma. Panels **a** the peptide-to-protein counts of plasma maintained frozen or on ice; **b** the peptide-to-protein distribution of plasma incubated at room temperature

**Fig. 8:**
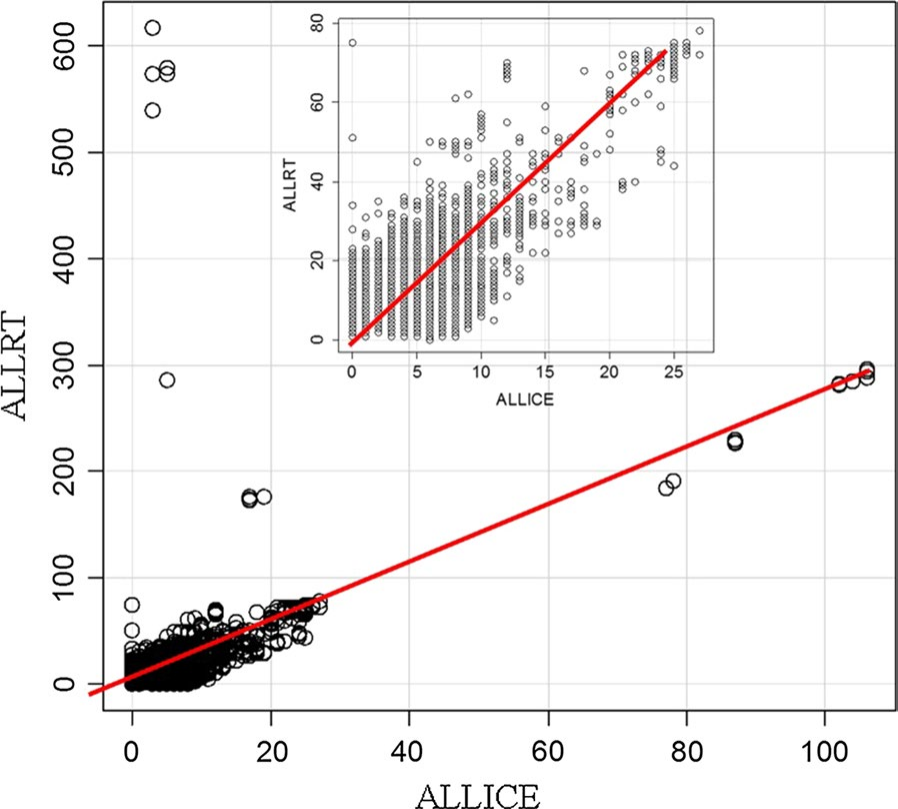
Regression analysis of the peptide-to-protein counts from samples incubated at room temperature (ALLRT) versus samples maintained on ice or frozen (ALLICE). Residual standard error: 2.945 on 22853 degrees of freedom, Adjusted R^2^: 0.3103, F statistic: 1.028e + 04 on 1 and 22853 DF, p-value < 2.2e − 16. To avoid infinity values 1 was added to each value (p1)

Samples maintained on ice showed proteins with up to a hundred correlated peptides after filtering (i.e. peptides to proteins) (Fig. 7a) but samples at room temperature showed greater numbers of correlations of peptides to proteins (Fig. 7b). Taking the ratio of room temperature (ALLRT) versus ice samples (ALLICE) showed more than one-hundred-fold variation of individual proteins with incubation at room temperature (Fig. 8). The proteins that showed the largest release of peptides at room temperature compared to ice was complement chain 4B (C4B) and C3 [2, 3]. Regression analysis revealed that most plasma proteins show on average a ~ twofold increase in sampling frequency at room temperature (Fig. 8).

### X!TANDEM analysis of endogenous tryptic peptides

**Fig. 9:**
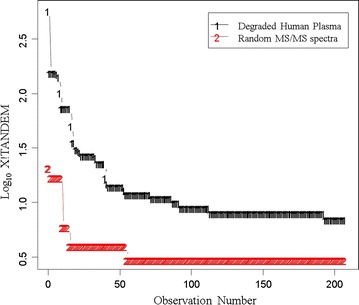
The log_10_ frequency of MS/MS correlations by X!TANDEM from authentic endogenous tryptic peptides from 563,095 MS/MS spectra collected from EDTA plasma versus the frequency from an equal number of random MS/MS spectra for the estimation of type I error rate in the proteins identified by Chi Square. The corrected mean peptide-to-protein counts of the first 200 protein accessions from X!TANDEM are shown. Note the peptide-to-protein distribution of real spectra exceeds that of random spectra by ≥ order of magnitude at all values and the Chi Square test indicates that the probability that the experimental data is the same as the null random model is essentially zero (p < 0.0001) and is a formal estimate of the type I error with respect to a null random model for the whole experiment using the classical statistical approach. The result indicates that the X!TANDEM algorithm that fits MS/MS spectra to predicted peptide spectra shows an acceptable type I error rate over the whole experiment

The results of the X!TANDEM algorithm were collected in SQL Server and analyzed compared to those of computer generated random MS/MS spectra or noise spectra (not shown) with the R statistical system that demonstrated a low probability of false positive identification (Fig. 9).

**Fig. 10:**
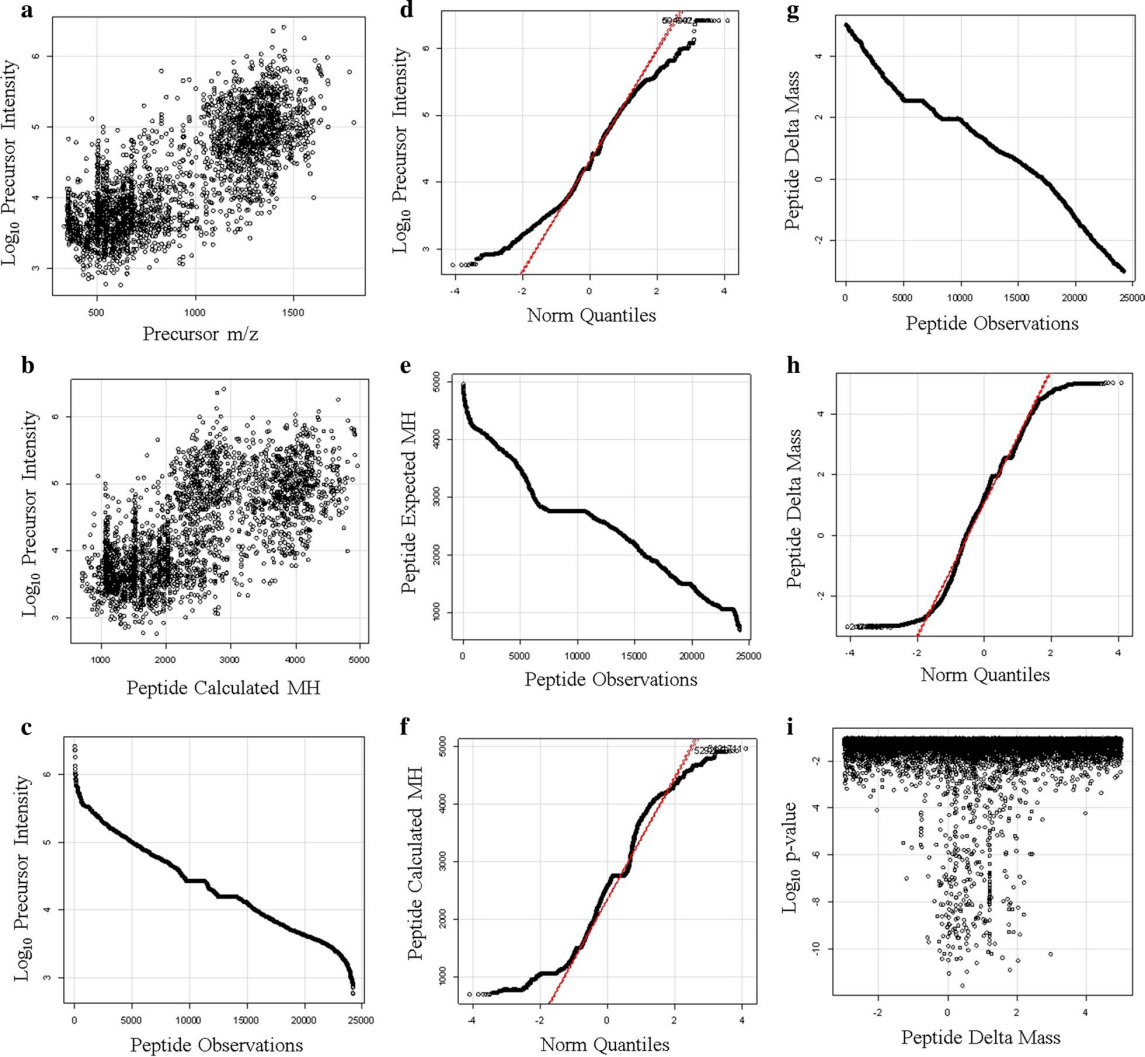
The distributions of the endogenous peptides from proteins with three or more independent correlations to the same protein accession by X!TANDEM. Panels **a** the scatter plot of log_10_ precursor intensity values versus precursor m/z; **b** the scatter plot of the log_10_ distribution of precursor intensity values versus peptide mass [M + H]^+^; **c** the sorted distribution of log_10_ intensity values; **d** the quantile plot of precursor log_10_ intensity values; **e** the sorted distribution of calculated peptide mass [M + H]^+^; **f** the quantile plot of peptide [M + H]^+^; **g** the sorted distribution of the peptide delta mass; **h** the quantile plot of the delta mass; **i** the relationship between delta M + H versus peptide log_10_ p-value

Most of the MS/MS spectra data was collected at values ranging from 350 to about 1700 m/z (Fig. 10a). The log_10_ distribution of precursor intensity increased with mass (momentum) as expected from impact detection (Fig. 10b); About 24,000 precursors with intensity values ranging from E3 to E6 arbitrary detector counts were observed (Fig. 10c). The quantile plot of the log_10_ distribution of precursor intensity values shows that after log_10_ transformation that intensity approaches Gaussian normality (Fig. 10d). The calculated peptide [M + H]^+^ values ranged from 1000 to 5000 Da (Fig. 10e). Moreover, the quantile plot strongly indicated that peptide [M + H]^+^ was sampled from a Gaussian (i.e. normal) population (Fig. 10f). The peptide delta mass values ranged from − 2  to + 4 Da consistent with the known error of the instrument and the presence of natural isotopes (Fig. 10g). The quantile plot of the delta mass values was apparently Gaussian with a mean of + 1, i.e. showed a normal statistical distribution with about 68% of the peptides within 1 Da of the mean predicted [M + H]^+^ while 95% were within 2 Da of the mean consistent with the presence of natural isotopes (Fig. 10h). The relationship between delta M + H versus peptide p-value was also apparently Gaussian (Fig. 10i).

**Fig. 11:**
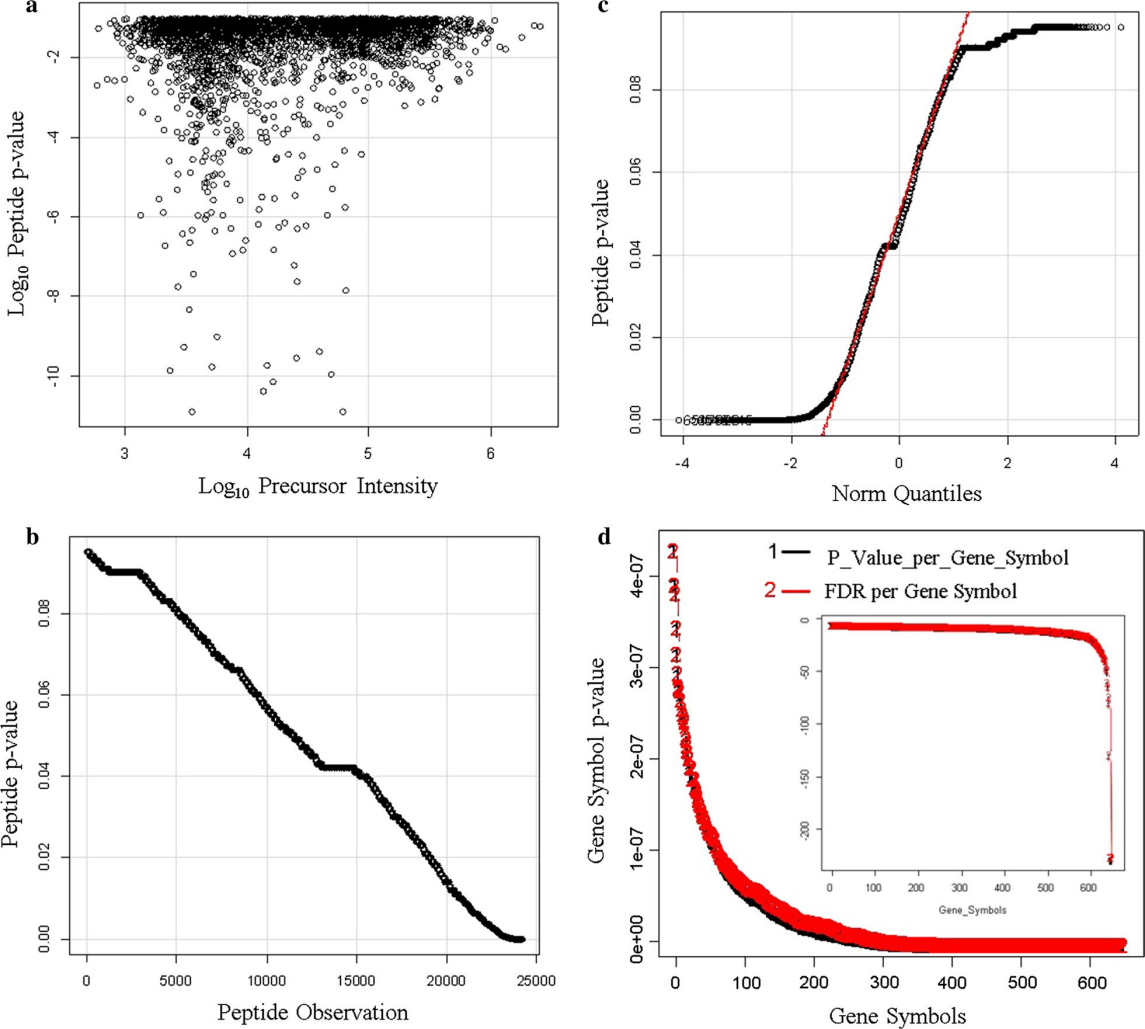
The confidence of endogenous tryptic peptides and proteins identified by X!TANDEM from human plasma on ice and incubated at room temperature. The X!TANDEM algorithm was used to assign the probability of type I error to peptides and thus the cumulative probability of type I error of proteins with 3 or more independent peptide correlations. Panels **a** scatter plot of peptide Log_10_ p-value versus Log_10_ precursor intensity; **b** sorted peptide p-values versus observations; **c** Peptide p-value quantile plot; **d** cumulative protein p-value per protein accession {Black Gene Symbol p-value, Red, FDR (false discovery rate) computed by the method of Benjamini and Hochberg [37]}

There was no relationship between log p-value and the peptide intensity for precursors ≥ 1000 (E3) counts (Fig. 11a). The sorted p-values showed a linear relationship with an average p-value of about 0.05 (Fig. 11b) that was normally distributed (Fig. 11c). Computing the cumulative p-value for proteins with 3 or more peptides showed that the protein Gene Symbol p-values ranged from E-7 to E-200 (Fig. 11d).

## Discussion

The aim of this study was to perform the random and independent sampling of human plasma on ice versus room temperature to identify and quantify the peptides and proteins that were preferentially cleaved in normal human plasma during incubation at room temperature. In the case of HPLC we may consider that each peptide elutes in one Gaussian peak. The peptides may be sampled multiple times as they elute from the column and may not necessarily be sampled at the top of the Gaussian peak but randomly across the peak. Thus, the log_10_ precursor intensity values of LC–ESI–MS/MS approximate a Gaussian distribution [27, 28]. The instrument samples all precursors by MS and then samples the 5 most intense by MS/MS every second or two across the chromatogram. The instant that the data is sampled is random with respect to the starting time of each chromatogram in the automatically repeating sample cycle of 1 MS and then 5 MS/MS. The timing of the MS and MS/MS sampling cycles are essentially random across the replicate chromatography traces with respect to the start of the experiment and so the ions sampled in one replicate are independent from the next. Once the peptide peak passes it does not typically return and so the precursors in the HPLC effluent are sampled without replacement. Taken together then, the precursor intensity values from LC–ESI–MS/MS have been randomly and independently sampled without replacement from a Gaussian distribution and therefore may be analyzed by ANOVA. In contrast, the peptide counts approximate a Gamma distribution and may be analyzed by the Chi Square test.

### Biochemical methods

The consistent SDS-PAGE banding patterns showing no change in the major plasma proteins indicates that many of the well known plasma proteins are relatively stable. In contrast, lower total protein levels, and a concomitant increase in alcohol soluble amines, early in the incubation at room temperature were both consistent with the degradation of a pool of proteins in plasma with time. The degradation of fresh human plasma itself was not reflected by changes in the banding patterns that were readily detectable by SDS-PAGE and this clearly rules out the presence of general proteases that visibly act on the many major plasma proteins with brief periods at room temperature [2, 47]. Moreover the stable banding pattern at room temperature observed here indicates the changes in SDS-PAGE banding patterns previously observed between different disease states were not merely due to differences in sample handling between normal and disease samples but rather must have resulted from the action of disease-specific protease activities [2, 47].

### Comparison of correlation parameters and algorithms

The agreement on the set of proteins identified between no enzyme specified (that shows a large degree of freedom and might fit the MS/MS spectra to any human peptide sequence), versus the proteins identified by fully tryptic peptides, is powerful evidence in favour of the veracity and low type I error of MS/MS correlation with an ion trap. The close agreement between no enzyme and fully tryptic correlations that both show a similar small set of proteins with multiple peptides, while the vast majority of human protein sequences show no correlation, is entirely consistent with the high fidelity of MS/MS spectra matching to predicted fragmentation patterns as estimated from the agreement of blood proteins between groups [31, 48], the computation of p-values from the fit of MS/MS spectra [30] or from first principles using a null random model [23, 24, 26–28, 30] and recently confirmed by the elaborate synthesis of 300,000 peptides [51]. With respect to fully tryptic peptides, the MASCOT, OMSSA, X!TANDEM and SEQUEST algorithms all agreed on the identity of the most frequently sampled peptides of EDTA plasma indicating that all the algorithms can identify the major plasma peptides. In agreement with previous results [23, 24], the endogenous tryptic peptides identified by X!TANDEM with at least three independent peptides also showed a low Type I (false positive) error rate after comparison to computer generated random (and noise) spectra consistent with the low cumulative protein p-values computed here. Conversely, MASCOT and OMSSA failed to identify many of the authentic peptides credibly detected by X!TANDEM and thus suffered from a high Type II (false negative) error rate under our conditions. The failure of MASCOT and OMSSA to identify many proteins may result from the heuristic nature of these algorithms that rely on arbitrary composite scoring schemes that include precursor mass accuracy and the fit of the MS/MS spectra relative measures instead of accepting the best match of the MS/MS data based on correlation coefficient or goodness of fit. Thus, MASCOT and OMSSA failed to meet the basic condition of providing the simplest model that explains all of the data. In contrast, SEQUEST that meets the obligate requirement to provide the simplest model that explains all data, frequently identified Titin and other giant proteins at a low frequency that likely results from the near random distribution of mis-correlations and so can be directly corrected by running noise or random MS/MS spectra simulations [23, 24, 26–28]. Thus for C18 solid phase extraction of endogenous peptides and micro scale LC–ESI–MS/MS of EDTA plasma with a simple linear ion trap, X!TANDEM was the best choice of algorithm to fit the MS/MS spectra without correction by random or noise spectra [29].

### Baseline EDTA Plasma model

Collecting EDTA plasma directly onto ice and processing ice cold may be a practical way to avoid cellular or biochemical reactions that might vary over time and thus create pre-analytical variation. Here a solid baseline was established for the EDTA plasma by incubating on ice or ice plus inhibitors over a time compared to plasma incubated at room temperature. Many proteins showed about a two fold variation between ice and room temperature samples consistent with the degradation of plasma proteins over time and sample handling conditions [2, 5–21]. Collecting the EDTA plasma samples directly onto ice demonstrates that, in contrast to many reports, the complement peptides are almost non-observable in the baseline EDTA plasma but are almost instantaneously released at room temperature. From this study it is clear that EDTA plasma may be collected and processed ice cold to avoid the rapid degradation of a pool of proteins that commences rapidly upon incubation at room temperature.

### X!TANDEM analysis

The tryptic peptides identified by X!TANDEM show a low type I error rate and are not heavily contaminated with mis-correlations to TTN or other giant proteins [23, 24, 27, 28]. The results of the X!TANDEM algorithm showed that the proteins identified by ≥ 3 tryptic peptides with intensity values ≥ E3 counts with a linear 2D quadrupole ion trap show a negligible type I error in agreement with previous studies [23, 26, 27, 30, 31]. The peptide fit by X!TANDEM reasonably explained the observed MS/MS fragmentation [2, 29, 52] and showed log intensity, log p-value and delta mass that were Gaussian and so were ideal for statistical analysis. From the computed p-values and the comparison to random spectra we can unambiguously conclude that plasma proteins identified with at least three peptides by X!TANDEM are statistically reliable enough for further study (~ E-3 to E-300). For simplicity, the peptides identified from SEQUEST that map to the proteins identified by X!TANDEM will reasonably meet the obligation to provide the simplest model to explain all data while avoiding type I error.

### False discovery rate

The recent paper of Zolg et al. with 300,000 synthetic peptides confirmed the observed MS/MS spectra closely match those of the predicted MS/MS spectra [51]. The residues of most amino acids (except Ile vs Leu 113 m/z and Gln vs. Lys 128 m/z (d 0.05)) can be resolved by the ion trap within 0.5 Da in the MS/MS but some high resolution mass spectrometers do not in practice distinguish these much better. While there may be some peptides that cannot be definitively discriminated by MS/MS with an ion trap there remain many that can and thus serve as the basis of a practical technology for identifying and quantifying peptides and proteins [53–56]. Thus multiple peptides from many proteins can be sensitively assigned using the robust ion trap with confidence using the MS/MS spectra. The X!TANDEM algorithm emphasizes the fit of the tandem MS/MS spectra within 0.5 Da from precursors ± 3 m/z [29, 52]. As previously demonstrated in a direct comparison of authentic MS/MS spectra to false positive noise or random MS/MS spectra: the p-value of the peptide fits cannot be effectively used to separate authentic data from false positive data. In contrast, the high frequency of peptide assignment to the small set of highly-sampled proteins efficiently separates authentic data from false positive noise and random data that is evenly distributed across the database at a low frequency [23, 24, 26–28]. It has been unambiguously and clearly demonstrated that the frequency of peptide correlation to proteins efficiently distinguishes authentic results from false-positive noise or random spectra results [23, 24, 27]. The computation of the False Discovery Rate by the method of Benjamini and Hochberg [37] agreed with null random models of noise and random spectra that the identification of proteins from multiple peptides showed a low type I error rate.

### The cleavage products of EDTA plasma

The comparison of samples on ice versus room temperature shows that thousands of proteins may be directly detected and monitored from plasma by solid phase extraction with C18 peptide collection and robust analysis with micro electrospray. The experiment demonstrates it is feasible to compare plasma samples across experimental treatments using the endogenous peptide frequencies without extensive chromatographic pre-separation of proteins. The complement proteins such as C4 and C3 are in high abundance in blood and designed to be rapidly processed in response to biochemical signals that might be propagated via the action of kininogen [57, 58]. In contrast, fibrinogen peptides, that are abundant in serum [2], did not show highly elevated levels of peptides in plasma at room temperature where no clotting occurs in agreement with the theory.

## Conclusion

Independent protein assays, free amine assays and LC–ESI–MS/MS methods all agree that a pool of proteins start to degrade soon after incubation of plasma samples at room temperature [2]. LC–ESI–MS/MS of tryptic peptides was more sensitive than SDS-PAGE, protein assays or free amine assays for the detection of the ex vivo proteolytic degradation of plasma. The majority of blood peptides show a fairly robust two-fold increase in the random sampling frequency with incubation at room temperature indicating that most of the peptides observed were ex vivo artefacts. It was feasible to collect plasma samples from clinical subjects and identify the endogenous tryptic peptides, and thus proteins, with simple C18 solid phase extraction for identification and quantification by micro electrospray LC–ESI–MS/MS with a linear ion trap. Here we show for the first time that random and independent sampling of endogenous blood peptides results in a population of thousands of peptides from hundreds of proteins that show intensity, calculated mass and delta mass values that are normally distributed and have a low type I error rate based on comparison to a null model of random spectra or noise and the fitting of the MS/MS spectra by the rigorous X!TANDEM algorithm that directly generates a p-value from the goodness of fit of the MS/MS spectra within 0.5 Da [29, 52]. The sampling frequency of peptides by LC–ESI–MS/MS may be used to detect variation over experimental treatments. Since the observed peptide frequency may vary by about two with respect to ice cold controls, it follows that in general, differences between disease states or experimental treatments would have to exceed the threshold of twofold enrichment in order to rule out confounding effects from sample handling.

## Additional file

**Additional file 1.** DHP pass 17 distinct protein matrix.

## Abbreviations

ACN: acetonitrile
CBBR: coomassie brilliant blue
SDS-PAGE: sodium dodecyl sulfate polyacrylamide gel electrophoresis
